# Tunable Pseudo-Piezoelectric Effect in Doped Calcium Titanate for Bone Tissue Engineering

**DOI:** 10.3390/ma14061495

**Published:** 2021-03-18

**Authors:** Abdullah Riaz, Kerstin Witte, Wiktor Bodnar, Hermann Seitz, Norbert Schell, Armin Springer, Eberhard Burkel

**Affiliations:** 1 Chair of Microfluidics, Faculty of Mechanical Engineering and Marine Technology, University of Rostock, Justus-von-Liebig-Weg 6, 18059 Rostock, Germany; hermann.seitz@uni-rostock.de; 2 INP Leibniz Institute for Plasma Science and Technology, Felix-Hausdorff-Str. 2, 17489 Greifswald, Germany; kerstin.witte@inp-greifswald.de (K.W.); wiktor.bodnar@inp-greifswald.de (W.B.); 3 Department of Life, Light and Matter, University of Rostock, Albert Einstein-Str. 25, 18059 Rostock, Germany; 4 Helmholtz-Zentrum Geesthacht, Max Plank-Str. 1, 21502 Geesthacht, Germany; norbert.schell@hzg.de; 5 Medical Biology and Electron Microscopy Centre, University Medical Center Rostock, Strempel-Str. 14, 18057 Rostock, Germany; armin.springer@med.uni-rostock.de; 6 Institute of Physics, University of Rostock, Albert Einstein-Str. 23-24, 18059 Rostock, Germany; eberhard.burkel@uni-rostock.de

**Keywords:** CaTiO_3_, doping, piezoelectric effect, X-ray diffraction, nanostructure, bone tissue engineering

## Abstract

CaTiO_3_ is a promising candidate as a pseudo-piezoelectric scaffold material for bone implantation. In this study, pure and magnesium/iron doped CaTiO_3_ are synthesized by sol-gel method and spark plasma sintering. Energy dispersive X-ray mapping confirm the homogenous distribution of doping elements in sintered samples. High-energy X-ray diffraction investigations reveal that doping of nanostructured CaTiO_3_ increased the strain and defects in the structure of CaTiO_3_ compared to the pure one. This led to a stronger pseudo-piezoelectric effect in the doped samples. The charge produced in magnesium doped CaTiO_3_ due to the direct piezoelectric effect is (2.9 ± 0.1) pC which was larger than the one produced in pure CaTiO_3_ (2.1 ± 0.3) pC, whereas the maximum charge was generated by iron doped CaTiO_3_ with (3.6 ± 0.2) pC. Therefore, the pseudo-piezoelectric behavior can be tuned by doping. This tuning of pseudo-piezoelectric response provides the possibility to systematically study the bone response using different piezoelectric strengths and possibly adjust for bone tissue engineering.

## 1. Introduction

It is well known that the human bone exhibits significant electrical activity for its growth and healing process [1,2]. Piezoelectric ceramics allow for the delivery of an electrical stimulus without the need for an external power source for biomedical applications [3]. Therefore, as for scaffolds in tissue engineering, there is an increasing interest in piezoelectric ceramics because of their potential for providing electrical stimulation to cells promoting tissue repair [3,4,5].

Piezoelectric ceramics used in medical applications such as bone tissue engineering have to be non-cytotoxic, whereas most common ceramics like zirconate titanate, lead titanate or their compounds with piezoelectric properties are harmful for humans upon their ion dissolution in biological fluids [6,7]. In contrast, perovskite calcium titanate (CaTiO_3_) ceramic is a non-cytotoxic compound. It was inadvertently found that non-piezoelectric CaTiO_3_ formed at the interface between hydroxyapatite (HA) and titanium implants during a coating procedure. This formation of CaTiO_3_ on titanium-coated HA increased the osteoblast adhesion [8]. Studies also show that the bone binding strength is improved due to the modification of titanium surface with CaTiO_3_ [9,10]. It could be possible that CaTiO_3_ can even lead to better results if it shows piezoelectric behavior. Recently, it was reported that distorted structure of spark plasma sintered CaTiO_3_ showed pseudo-piezoelectric behavior which most likely was generated by local instability (due to defects like oxygen vacancies) of the normal TiO_6_ octahedra [11]. Since the strength of piezoelectric effect can play an important role for triggering the bone cells in a healing process, there is a need to tune the pseudo-piezoelectric behavior of CaTiO_3_. This tuning of pseudo-piezoelectric response is necessary to systematically study the bone response using different piezoelectric strengths.

It should be possible to increase or tune the pseudo-piezoelectric behavior of CaTiO_3_ through slightly changing the crystal structure of CaTiO_3_ by doping [12]. Consequently, the pseudo-piezoelectric behavior can be tuned in a way that the bone cells can sense stimulations to improve the healing process. Utilizing doping elements which are already present in human body, i.e., Mg and Fe, should not cause any harm [13,14]. In the present study, pure CaTiO_3_ and doping with Mg and Fe were prepared and the effect of doping on the crystal structure and pseudo-piezoelectric behavior of CaTiO_3_ was investigated.

## 2. Materials and Methods

The pure, Mg and Fe doped CaTiO_3_ powders were prepared by sol-gel synthesis [15]. Calcium nitrate tetrahydrate (Ca(NO_3_)_2_·4H_2_O) powder (Chemsolute/Th. Geyer GmbH and Co. KG, Renningen, Germany), Magnesium nitrate hexahydrate (Mg(NO_3_)_2_·6H_2_O) and iron(III) nitrate nonahydrate (Fe(NO_3_)_3_·9H_2_O) powders (Alfa Aesar, Kandel, Germany), and titanium isopropoxide (Ti(OC_3_H_7_)_4_) (Sigma-Aldrich/Merck KGaA, Darmstadt, Germany) were used as precursors. The powders were prepared with the molar ratios of 1:1 of Ca:Ti and 9:10:1 of Ca:Ti:Fe/Mg, for pure and doped CaTiO_3_, respectively. Firstly, to prepare pure CaTiO_3_, Ca(NO_3_)_2_·4H_2_O and Ti(OC_3_H_7_)_4_ were dissolved separately in ethanol and then Ca(NO_3_)_2_·4H_2_O was mixed drop wise in Ti(OC_3_H_7_)_4_ solution. Afterwards, the solution was stirred for 24 h at room temperature. Secondly, to synthesize doped powders, Ca(NO_3_)_2_·4H_2_O along with (Mg(NO_3_)_2_·6H_2_O)/(Fe(NO_3_)_3_.9H_2_O) was mixed drop-wise in Ti(OC_3_H_7_)_4_ solution. These solutions were also stirred for 24 h at room temperature. The resulting solutions were dried separately with a mini spray dryer (Büchi Labortechnik AG, Essen, Germany) and also in an oven (Nabertherm GmbH, Lilienthal, Germany) at 180 °C for 2 h. Subsequently, the powders were calcined in an oven at 650 °C in air.

The calcined powders were densified using the HP D5 Spark Plasma Sintering (SPS) unit (FCT Systeme GmbH, Rauenstein, Germany) located in the Tycho Sinterlab, University of Rostock, Germany. The sintering of pure and doped powders was performed in vacuum of approximately 1 mbar. The applied load was adjusted to a value of 6 kN, resulting in a pressure of approximately 76 MPa inside of the die. The powders were heated up to 1025 °C (pure CaTiO_3_), 975 °C (Mg doped) and 925 °C (Fe doped) with a heating rate of 100 K/min. The samples were sintered for 5 min at elevated temperatures and afterwards cooled down naturally. To adjust the height of the pure and doped samples for further experiments, a diamond band saw was used to precisely cut the samples. The final dimensions of all samples were 10 mm × 2 mm (diameter × height).

For elemental mapping, samples were mounted on Al-SEM-carrier with adhesive conductive carbon tape (co. PLANO, Wetzlar, Germany) and coated with carbon under vacuum (EM SCD 500, Co. Leica, Bensheim, Germany). Afterwards, samples were analyzed by a field emission scanning electron microscope (SEM, MERLIN^®^ VP Compact, Co. Zeiss, Oberkochen, Germany) equipped with an energy dispersive X-ray (EDX) detector (XFlash 6/30, Co. Bruker, Berlin, Germany). A representative area (218.1 µm × 158.1 µm) of the samples were analyzed (for quantification: 500,000 counts were recorded) and mapped for elemental distribution on basis of the EDX-spectra data by QUANTAX ESPRIT v.2.0. (Co. Bruker, Berlin, Germany) Microanalysis software.

High Energy X-ray Diffraction (HEXRD) experiments were carried out at the High Energy Material Science (HEMS) beamline P07b located at high brilliance synchrotron radiation storage ring PETRA III, DESY, Hamburg, Germany. The basic design parameters of the storage ring are an energy of 6 GeV and a current of 100 mA. The source of X-rays for HEMS was a 2 m long standard PETRA undulator. To select the radiation with a wavelength of λ = 0.1424 Å, an indirectly water cooled single bounce monochromator with a Si(220) Laue crystal was used [16,17]. The experiments were carried out in transmission Debye–Scherrer geometry and the distance between sample and detector was 1230 mm. The diffraction patterns were collected with a 2D-area PerkinElmer detector (Santa Clara, CA, USA).

The direct piezoelectric measurements were performed using a dynamic method. The measurement setup is a modified form of the one from Fukada and Yasuda [2]. This in-house setup consists of a metallic framework, which fixed the sample to be examined between two insulating corundum plates attached to a Piezo-Actuator (PA) type PICMA P-888.31, PI Ceramics, Lederhose, Germany, and frame. Using a RIGOL DG4062 function generator, Beijing, China, a sine voltage of 20 V was applied to the PA and converted into a dynamic mechanical load to the sample which was excited to oscillate at a frequency of 10 Hz. The alternating voltage produced by the sample was collected using an MMF M68D1 charge amplifier, Radebeul, Germany, with a gain of 1000 mV/pC. The data were collected with the help of PicoScope 6, Pico Technology, St. Neots, UK. This PicoScope was connected to a charge amplifier. First, a Non-Piezoelectric Material (NPM) was tested to interpret the maximum noise generated by the setup. Afterwards, pure and doped CaTiO_3_ were investigated.

## 3. Results and Discussion

**Figure 1:**
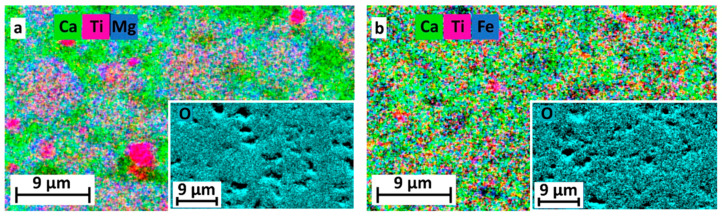
Elemental maps for the elements of the (**a**) Mg and (**b**) Fe doped CaTiO_3_. (See web version for colors).

Figure 1 shows the EDX elemental mapping of the Mg and Fe doped CaTiO_3_ samples, and the determined atomic fraction (at.%) of elements are presented in Table 1 together with pure CaTiO_3_. In pure CaTiO_3_, O, Ca and Ti are only present, but in doped samples, few at.% of Mg and Fe are also detected (Table 1). Additionally, a nearly homogenous distribution of these elements is found in the doped samples after SPS, with a strong reduction of large elemental segregation at the length scale of Figure 1. Carbon is also present in all samples (Table 1) which is caused by the sample’s preparation for EDX experiments.

**Figure 2:**
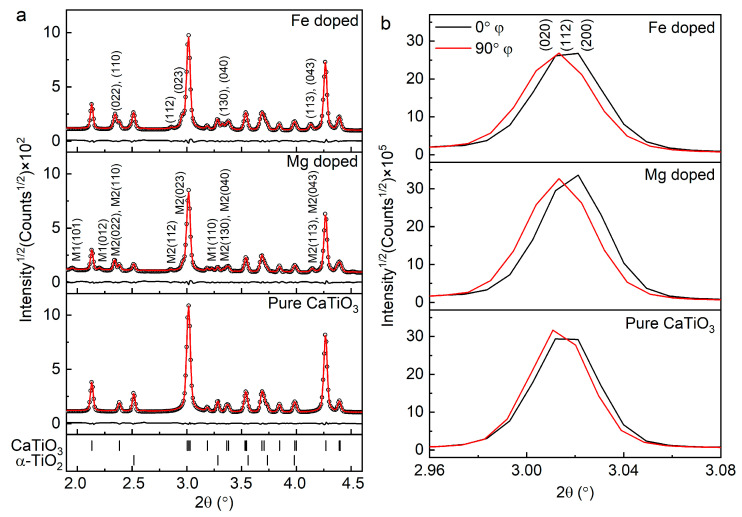
High Energy X-ray Diffraction (HEXRD) patterns of the pure, Mg and Fe doped CaTiO_3_. The experimental pattern (black dots), the fitted pattern (red line) and the differential pattern (black line) are presented. Furthermore, Bragg positions of CaTiO_3_ and α-TiO_2_ phases are shown and main peaks of MgTiO_3_ (M1), MgTi_2_O_5_ (M2) and Fe_2_TiO_5_, are labelled by corresponding Miller indices (**a**). Main peaks of CaTiO_3_ phases of the pure, Mg and Fe doped samples at 0° and 90° azimuthal sectors. The peak is labeled by corresponding Miller indices (**b**). (See web version for colors).

HEXRD patterns were integrated into 5° azimuthal steps and then all patterns were refined by Rietveld refinement [18] using the MAUD program v.2.94. (written by Luca Lutterotti, University of Trento, Italy [19,20]. It can be seen in Figure 2a that in all diffraction patterns, the main orthorhombic (pbnm) phase of nanostructured CaTiO_3_ is present and their crystallite sizes (CS) are presented in Table 1. Small amounts of MgTiO_3_ and MgTi_2_O_5_ phases were found in the Mg doped sample. However, in the Fe doped sample, only an additional Fe_2_TiO_5_ phase was present. The weight fractions of the corresponding phases are also presented in Table 2. Moreover, significant changes in the lattice parameters of CaTiO_3_ orthorhombic phase of the doped samples are observed compared to the pure CaTiO_3_ (see supplementary). It could be an indication of the replacement of doped cations, i.e., Mg and Fe by Ca cations, in CaTiO_3_ crystal structure [21].

Besides the integration of HEXRD patterns of whole Debye–Scherrer rings, the rings were also partially integrated into every 85° azimuthal step with a step width of 5° (see supplementary). In Figure 2b, the main peaks of orthorhombic phase of CaTiO_3_ are presented with Miller indices (020), (112) and (200) at 0° and 90° azimuthal sectors. It is noticeable that the main peak position of pure CaTiO_3_ at 90° azimuthal sector is shifted from 0° azimuthal sector. This peak shift is due to the lattice distortions influenced by SPS [22]. Furthermore, the peak position in Mg and Fe doped samples are shifted even more compared to pure CaTiO_3_ indicating further lattice distortions as a result of doping elements. These lattice distortions and strains in the host lattice are due to cations replacement at the A site of perovskite and have also been observed previously [12,21].

To look over into more detail on the crystal structures of pure and doped samples, the lattice strains (ε) were investigated as a function of azimuthal sectors. For that purpose, the lattice parameters at the individual azimuthal sectors, i.e., 0°, 90°, 180° and 270°, were determined and extracted by Rietveld refinement [18], and afterwards calculated with the help of Equation (1) [11]:

ε = (q − q_o_)/q_o_ (1)

where q represents lattice parameter values at azimuthal sectors when they are considered as an initial point, for example 0°, 90°, 180° and 270° and q_o_ represents lattice parameter values at azimuthal sectors when they are considered as a final point, for example 90°, 180°, 270° and 0°.

**Figure 3:**
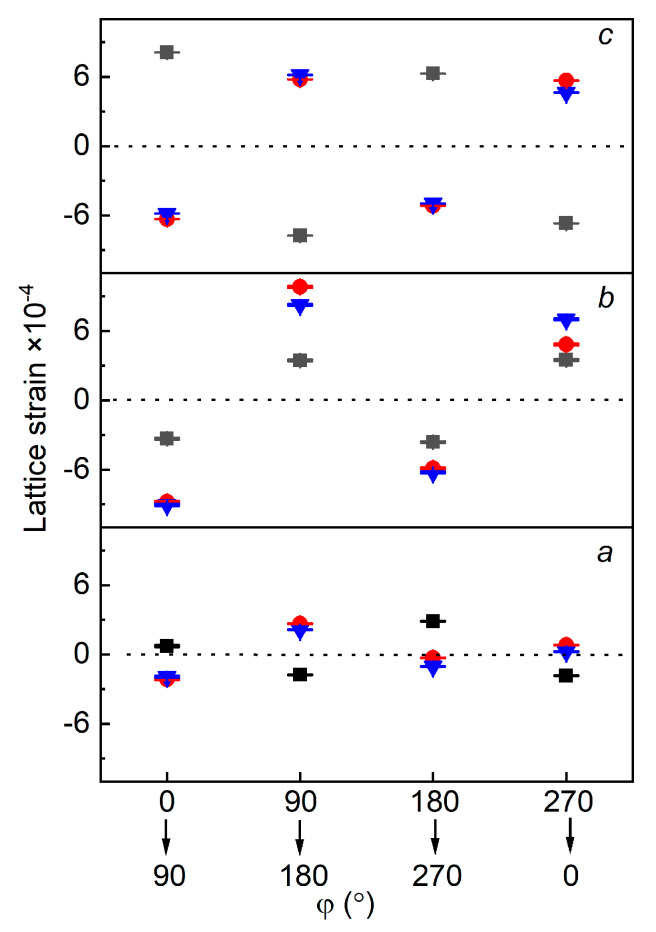
Lattice strain of lattice parameters *a*, *b* and *c* of main orthorhombic CaTiO_3_ phases of the pure (black color), Mg (blue color) and Fe doped (red color) samples vs azimuthal sector. (See web version for colors).

Figure 3 shows the lattice strain of lattice parameters *a*, *b* and *c* of main orthorhombic CaTiO_3_ phase as a function of initial to final azimuthal sectors. In the pure CaTiO_3_ sample, maximum lattice strain due to structural distortions (caused by defects) is observed in the lattice parameter *c*, and lattice strain in lattice parameters *a* and *b* is also present. This lattice strain is influenced by SPS, as reported in [11]. These azimuthal sectors dependent lattice strain in lattice parameters show that the Debye–Scherrer rings are elliptical but not circular in shape which is caused by structural distortions. Hence, electric dipole moments were generated because of the shift of positive and negative charges and resulted in the pseudo-piezoelectric effect [11], whereas in the Mg and Fe doped CaTiO_3_, maximum lattice strain is observed not only in the lattice parameter *c*, but also in *b*. The magnitude of lattice strain for *a* remains approximately constant compared to pure CaTiO_3_. It could be interpreted as stronger structural distortions (more elliptical Debye–Scherrer rings) and more defects in doped samples. For example, defects can be further influenced by the replacement of Ca to Mg or Fe cations which led to a change in the distortion of the CaTiO_3_ crystal structure and may strengthen the generated electric dipole moments. These increased crystal distortions due to cations replacements should also influence the piezoelectric behavior and tune the strength of the piezoelectric effect.

**Figure 4:**
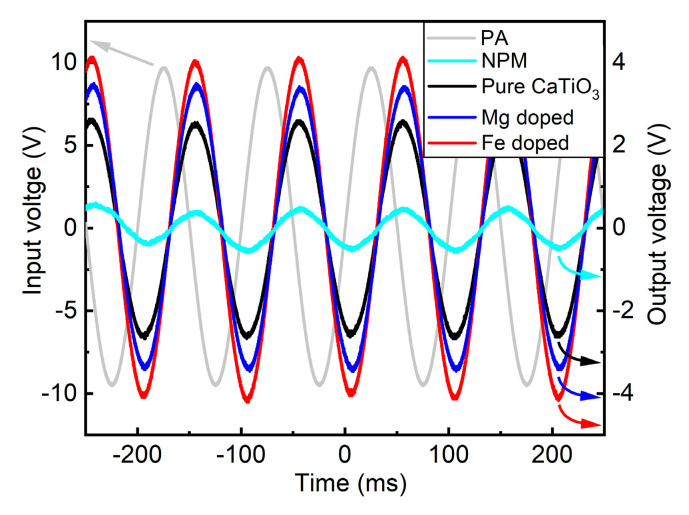
Time dependent voltage measurement of the NPM as well as pure, Mg and Fe doped CaTiO_3_ though dynamic method. Moreover, the input voltage of the PA is shown. (See web version for colors).

Figure 4 shows the responses of NPM and all sintered samples measured by the direct piezoelectric effect. The alternating output voltages generated by the pure, Mg and Fe doped CaTiO_3_ can be seen. Furthermore, the input voltage represents the voltage which was applied for the oscillation of PA. It appears that NPM (V_NPM_) produced a small alternating output voltage. This voltage represents the maximum noise generated by the experimental setup. It is conspicuous that the alternating output voltage (V_CaTiO_3__) of the pure CaTiO_3_ is significantly larger than the maximal noise of the setup. A further increase in V_CaTiO_3__ of the Mg doped sample is observed, and V_CaTiO_3__ of the Fe doped sample is even larger. It indicates the enhanced electric dipole strength of doped samples possibly caused by the different ionization states and radii of replaced Mg and Fe cations, and their influence on the increased structural distortions [23]. As a result, it also increases the pseudo-piezoelectric strength. L.H. Oliveira et al. also reported structural distortions as a function of Mg doping which was associated to the different ionic radii of cations and their repulsion in the A site of the perovskite structure [21]. It could also be possible that the small quantity of additional phases in doped samples slightly influenced the piezoelectric behavior. To calculate the maximal charge (Q) produced by all samples using the direct piezoelectric effect, equation 2 was used [11] and the values are shown in Table 2. Considering the increase in the pseudo-piezoelectric strength, it would be interesting to study the influence of additional phases, dependence of the fraction of doping elements in CaTiO_3_ and their crystallite sizes for further application as a bone scaffold material. Additionally, the pseudo-piezoelectric response of the material can be tuned by doping giving the opportunity to study its impact in bone tissue engineering.

*Q*_CaTiO_3__ = V_CaTiO_3__/Gain − V_NPM_/Gain (2)

## 4. Conclusions

In this study, pure, Mg and Fe doped CaTiO_3_ were synthesized by sol-gel method and SPS and pseudo-piezoelectric strength was successfully tuned by doping of Mg and Fe in CaTiO_3_. The EDX-mapping showed the homogenous distribution of doping elements in sintered samples. The significant changes in lattice parameters of orthorhombic CaTiO_3_ phase in pure and doped samples were observed. The doping of nanostructured CaTiO_3_ increased the strain and defects in the structure of CaTiO_3_ compared to the pure sample. Hence, an increase in pseudo-piezoelectric response is also observed in doped CaTiO_3_. This effect was determined using the direct piezoelectric method. The produced charge in Mg doped sample was larger than pure CaTiO_3_ and it was even more in Fe doped CaTiO_3_. This tuning of pseudo-piezoelectric behavior provides the possibility of studying the bone response on a different pseudo-piezoelectric strength of scaffold material and to be engineered accordingly. This investigation can be extended to studying additional phases and whether they show piezoelectric behavior or not, as well as different fraction of doping elements in CaTiO_3_ and their influence on piezoelectric behavior for further application as a bone scaffold material.

## Figures and Tables

**Table 1:** Determined atomic fraction (at.%) of elements in the pure, Mg and Fe doped CaTiO_3_. The standard deviation of each measurement is also presented.

CaTiO_3_	Composition (at.%)					
	O	Ca	Ti	Mg	Fe	C
Pure	57 ± 5	16 ± 1	19 ± 1	–	–	8 ± 1
Mg doped	57 ± 5	15 ± 1	18 ± 1	2 ± 0	–	8 ± 1
Fe doped	56 ± 5	14 ± 1	17 ± 1	–	2 ± 0	10 ± 1

**Table 2:** Determined crystallite sizes (CS), weight fractions (wt.%) of the contributing phases, and maximal charge produced by the pure, Mg and Fe doped CaTiO_3_. The error of each measurement is also presented.

CaTiO_3_	CS (nm)	Phases (wt.%)					Q (pC)
	CaTiO_3_	CaTiO_3_	α-TiO_2_	Fe_2_TiO_5_	MgTiO_3_	MgTi_2_O_5_	
Pure	219 ± 7	94.8 ± 0.5	5.1 ± 0.3	–	–	–	2.1 ± 0.3
Mg doped	175 ± 8	86.2 ± 0.2	4.7 ± 0.4	–	4.8 ± 0.3	4.2 ± 0.2	2.9 ± 0.1
Fe doped	163 ± 4	89.3 ± 0.4	5.2 ± 0.1	5.4 ± 0.2	–	–	3.6 ± 0.2

## Data Availability

The data presented in this study are available on request from the corresponding author.

## Supplementary Materials

The following are available online at https://www.mdpi.com/1996-1944/14/6/1495/s1, Figure S1: Lattice parameters of orthorhombic phase of the pure, Mg and Fe doped CaTiO_3_, Figure S2: The 2D diffraction image shows Debye-Scherrer rings collected from HEXRD experiment. The azimuthal sectors at 0°, 90°, 180° and 270° are presented with red areas.
