# Supplementary material for: Tunable Pseudo-Piezoelectric Effect in Doped Calcium Titanate for Bone Tissue Engineering
Source: Materials (Basel). 2021 Mar 18;14(6):1495. doi: 10.3390/ma14061495 (PMC8003264; doi:10.3390/ma14061495)
Supplement: Supplementary file 1 [file materials-14-01495-s001.pdf]

materials

MDPI

Supplementary Materials

Tunable Pseudo-Piezoelectric Effect in Doped Calcium Titanate for Bone Tissue Engineering

Abdullah Riaz [1,*], Kerstin Witte [2], Wiktor Bodnar [2], Hermann Seitz [1,3], Norbert Schell [4], Armin Springer [5] and Eberhard Burkel [6]

[1] Chair of Microfluidics,Faculty of Mechanical Engineering and Marine Technology, University of Rostock, Justus-von-Liebig-Weg 6, 18059 Rostock, Germany; hermann.seitz@uni-rostock.de
[2] INP Leibniz Institute for Plasma Science and Technology, Felix-Hausdorff-Str. 2, 17489 Greifswald, Germany; kerstin.witte@inp-greifswald.de (K.W.); wiktor.bodnar@inp-greifswald.de (W.B.)
[3] Department of Life, Light and Matter, University of Rostock, Albert Einstein-Str. 25, 18059 Rostock, Germany
[4] Helmholtz-Zentrum Geesthacht, Max Plank-Str. 1, 21502 Geesthacht, Germany; norbert.schell@hzg.de
[5] Medical Biology and Electron Microscopy Centre, University Medical Center Rostock, Strempel-Str. 14, 18057 Rostock, Germany; armin.springer@med.uni-rostock.de
[6] Institute of Physics, University of Rostock, Albert Einstein-Str. 23-24, 18059 Rostock, Germany; eberhard.burkel@uni-rostock.de
* Correspondence: abdullah.riaz@uni-rostock.de; Tel.: +49-381-498-9138

Citation: Riaz, A.; Witte, K.; Bodnar, W.; Seitz, H.; Schell, N.; Springer, A.; Burkel, E. Tunable Pseudo-Piezoelectric Effect in Doped Calcium Titanate for Bone Tissue Engineering. *Materials* **2021**, *14*, 1495. https://doi.org/10.3390/ma14061495

Academic Editor: Andrea Spagnoli

Received: 27 January 2021
Accepted: 16 March 2021
Published: 18 March 2021

Publisher's Note: MDPI stays neutral with regard to jurisdictional claims in published maps and institutional affiliations.

Copyright: © 2021 by the authors. Licensee MDPI, Basel, Switzerland. This article is an open access article distributed under the terms and conditions of the Creative Commons Attribution (CC BY) license (http://creativecommons.org/licenses/by/4.0/).

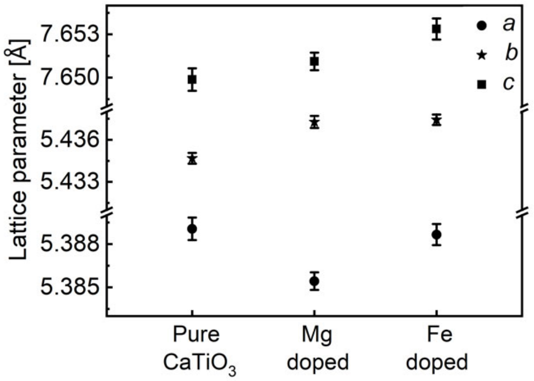

Figure S1. Lattice parameters of orthorhombic phase of the pure, Mg and Fe doped $CaTiO_3$.

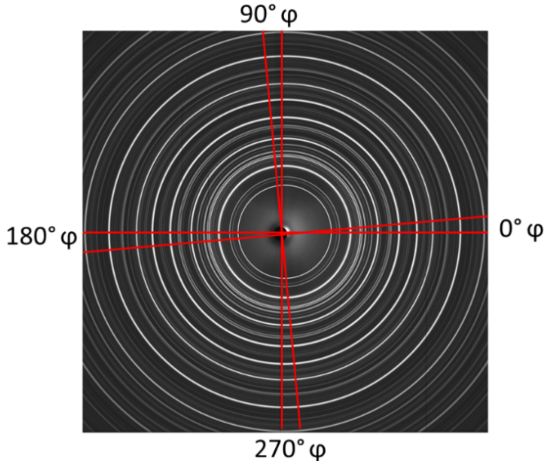

Figure S2. The 2D diffraction image shows Debye-Scherrer rings collected from HEXRD experiment. The azimuthal sectors at 0°, 90°, 180° and 270° are presented with red areas.
